# Dataset for assessing the efficiency factors in Malaysian ports: Dry bulk terminal

**DOI:** 10.1016/j.dib.2020.105858

**Published:** 2020-06-20

**Authors:** Norlinda Mohd Rozar, Muhammad Ashlyzan Razik, Mohamad Hazeem Sidik, Saadi Kamarudin, Mohd Rizal Ismail, Azman Azid, Rosni Othman

**Affiliations:** a Faculty Maritime Studies, University Malaysia Terengganu, Kuala Nerus, Malaysia; b Faculty of Entrepreneurship and Business, Universiti Malaysia Kelantan, Pengkalan Chepa, 16100 Kota Bharu, Malaysia; c Faculty Business and Mathematic, Help University, Malaysia; d Faculty Bioresources and Food Industry, Universiti Sultan Zainal Abidin, Besut Campus, 22200 Besut, Terengganu, Malaysia

**Keywords:** Normality test, Histogram, Dry bulk terminal, Efficiency

## Abstract

This research paper provides for the identification of dry bulk terminal efficiencies on the basis of 10 key performance factors in Malaysian ports. Data were collected from 18 dry bulk ports in Malaysia in 2017 through an online questionnaire and distributed via e-mail. The dispersion of the respondents corresponds approximately to the structure of the Malaysian maritime terminal in dry bulk. The data provides port management perceptions towards 10 variables that have been surveyed. Each perception assessed the level of efficiency factors based on a percentage rate of 100%. Efficiency factors in dry bulk terminals have been identified with varying characteristics based on a descriptive analysis table. The dataset presented consists of a brief analysis of all 10 variables involved, including the minimum, maximum, mean, interquartile median and standard deviation. In addition to the descriptive analysis, the normality test and histogram were also performed. Data can be used to measure ports-efficiency factors in another research.

Specifications table

**Table:** 

Subject	Strategy and Management			
Subject Area	Key drivers of Port Competitiveness			
More specific subject area	Dry Bulk Port Operation			
Type of data	Table, Graph and Figure			
How data was acquired	The data was collected from all of Malaysian Dry Bulk Terminal Ports using questionnaire distributed through email. From the primary sources, the data was digitised from corresponding archive.			
Data format	Raw, Filtered and analysed data			
Data collection parameters	Machines, Conventional labor oriented (CLO), Trucking efficiency minutes, Stockpile Locations			
Description of data collection	The data was collected from around of Malaysian ports at dry bulk terminal using an online questionnaire distributed to 18 Ports through email. The dispersion of respondents corresponds approximately to the structure of Malaysian maritime at the dry bulk terminal. The data provides full responses from the head of the port management. Each respond assessed the level of efficiency factors by percentage rate given from the total of 100%.			
Data source location	No.	Port	Region	Coordinate
	1	Sandakan	Sabah	5.8120° N, 118.0769° E
	2	Kota Kinabalu		5°58′60.00′'N 116°4′0.00′'E
	3	Kudat		6°52′60.00″N 116°50′60.00″E
	4	Labuan		5.2765° N, 115.2430° E
	5	Bintulu		3°16′0.00″N 113°4′0.00″E
	6	Tawau		4.2460° N, 117.8807° E
	7	Lahad Datu		5.0202° N, 118.3495° E
	8	Tanjung Manis	Sarawak	2.1575° N, 111.3391° E
	9	Kuching		1°33′13.76″N 110°20′7.00″E
	10	Sarikei		2°7′60.00″N 111°31′60.00″E
	11	Sibu		02°17′16″N 111°49′51″E
	12	Kuantan	Central	3.9767° N, 103.4242° E
	13	Kemaman	East Coast	4°24′58.48″N 103°15′18.02″E
	14	Johor	Southern	1.4438° N, 103.9064° E
	15	Penang	Northern	5.4098° N, 100.3679° E
	16	Lumut		4°13′0.01″N 100°37′0.01″E
	17	North		27.0442° N, 82.2359° W
	18	Westport	Western	2.9833° N, 101.4190° E
Data accessibility	https://data.mendeley.com/datasets/jxj6dt54w6/1			
Related research article	Rozar, N. M., Razik, M. A., & Sidik, M. H. M. (2018). The Factor Analysis of the Antecedents of Dry Bulk Terminal for Port Operation Improvement in Malaysia. International Journal of Engineering and Md. 10(6), 1801–1805.			

## Value of the data

• In dry bulk terminal, the data encapsulates a large number of Malaysian ports efficiency dataset.
• The data offers insight for assessing Malaysian Ports efficiency in dry bulk terminal where it can be used to comprehend the other terminals of Malaysian ports (e.g. changes in coastal shipping services and port facilities) into regional economic change; in the long run, give broad geographical and temporal coverage of the data.
• The data uncovers the variances of efficiency factors in dry bulk terminal ports and for port managers in order to build a long-term action strategy.

## 1 Data description

Table 2 shows the normality test from four different techniques, namely Kolmogorov-Smirnov and Shapiro-Wilk. The normality test was conducted from 10 variables as at Table 1. The result demonstrated that the dataset of Machines (VA.1), Conventional labor oriented (VA.2), Trucking efficiency < 15 min (VA.3), Trucking efficiency 15 – 30 min (VA.4), Trucking efficiency > 30 min VA.5). These are one of the facilities for Malaysians’ port managers to achieve higher level of efficiency in the port operation and it was categorised of cargo handling technology and equipment, and port information technology. Thus, affected in port trade to take initiatives to expand port capacity for trucking efficiency [1–2].

**Table 1:** Summary of the variable's descriptions.

Symbol	Descriptions
VA. 1	Machines
VA. 2	Conventional labor oriented
VA.3	Trucking efficiency < 15 min
VA.4	Trucking efficiency 15 – 30 min
VA.5	Trucking efficiency > 30 min
VA.6	Stockpile Locations < 1km
VA.7	Stockpile Locations 1 km – 3km
VA.8	Stockpile Locations 3 km – 5km
VA.9	Stockpile Locations 5 km – 10km
VA.10	Stockpile Locations > 10km

**Fig. 1:**
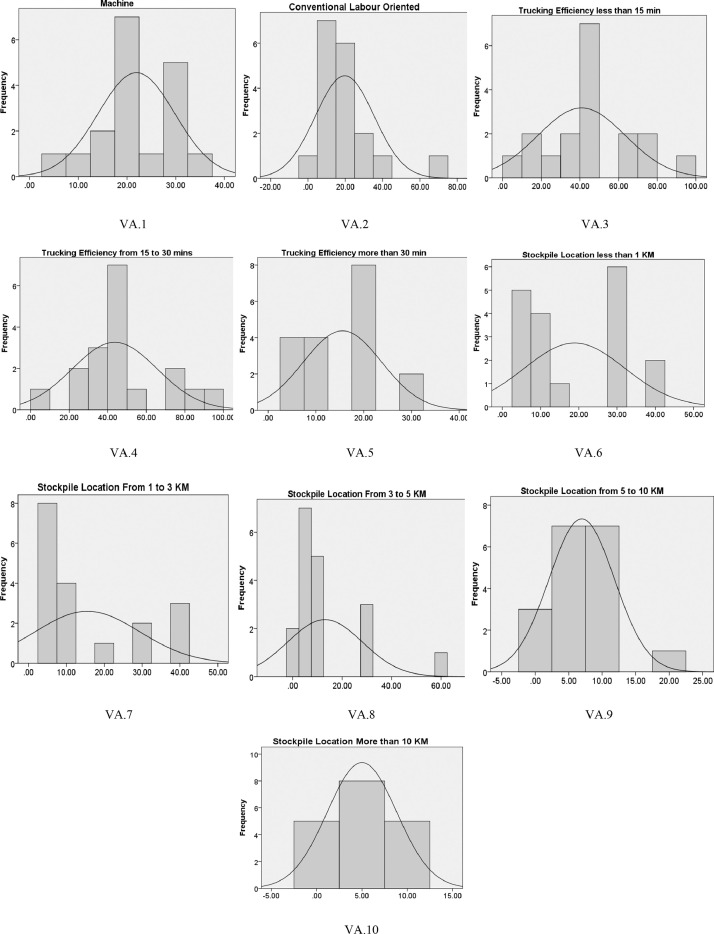
The normality test chart for port efficiency in dry bulk terminal.

**Fig. 2:**
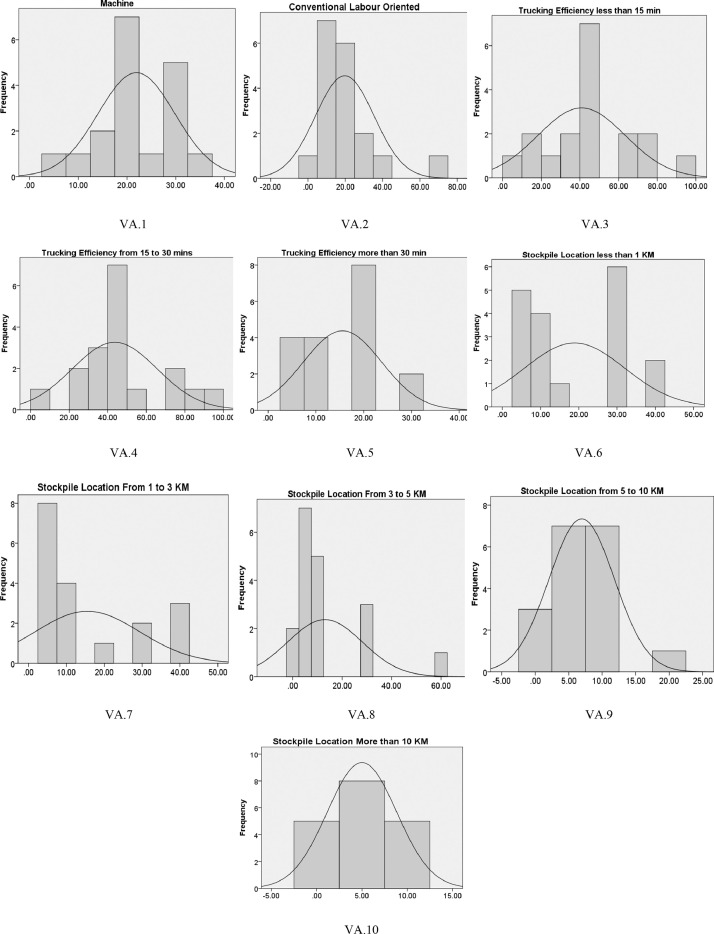
Histogram analysis for port efficiency in dry bulk terminal.

While, at Table 2 shows the normality test for Stockpile Locations as at Table 1**.** were consisted Stockpile Locations < 1 km (VA.5), Stockpile Locations 1 km – 3 km (VA.6) Stockpile Locations 3 km – 5 km (VA.7), Stockpile Locations 5 km – 10 km (VA.8), Stockpile Locations > 10 km (VA.9) are normal. Table 3 and Fig. 2 show the variability of all variables, i.e. the minimum, maximum, interquartile, median, mean standard deviation, Variance, skewness and Kurtosis. Figs. 1 and 2 show the normality test and histogram for each variable, respectively. The strategic location of a port significantly increases its efficiencies. From Fig. 1, the mean value for 18 ports are mostly equivalent for all types of variables. However, Stockpile Locations 5 km – 10 km (VA.10) consistently showed low value. The results were related with the position refers to of "diversion distance" concept where ships deviate from main trunk routes to the port. It was discussed by [3] said that the centrality of shipping routes is vital not only because it acts a port gateway but also as a hub for transhipment.

**Table 2:** Summary of the Case Processing Summary/ normality test.

	Tests of Normality						
		Kolmogorov-Smirnov^a^			Shapiro-Wilk		
Variables	Description	Statistic	df	Sig.	Statistic	df	Sig.
VA. 1	Machines	.128	18	.200*	.900	18	.058
VA. 2	Conventional labor oriented	.271	18	.001	.778	18	.001
VA.3	Trucking efficiency < 15 min	.237	18	.009	.938	18	.270
VA.4	Trucking efficiency 15 – 30 min	.287	18	.000	.903	18	.066
VA.5	Trucking efficiency > 30 min	.262	18	.002	.858	18	.011
VA.6	Stockpile Locations < 1km	.251	18	.004	.822	18	.003
VA.7	Stockpile Locations 1 km – 3km	.323	18	.000	.737	18	.000
VA.8	Stockpile Locations 3 km – 5km	.358	18	.000	.710	18	.000
VA.9	Stockpile Locations 5 km – 10km	.211	18	.034	.855	18	.010
VA.10	Stockpile Locations > 10km	.222	18	.019	.818	18	.003

⁎ This is a lower bound of the true significance.

a Lilliefors Significance Correction.

**Table 3:** Descriptive analysis of Demographic factors in dry bulk terminal for port efficiency.

		Statistic	Std. Error
VA.1	Mean	524.7222	80.38058
	95% Confidence Interval for Mean	Lower Bound	355.1340
		Upper Bound	694.3104
	5% Trimmed Mean	494.6358	
	Median	475.0000	
	Variance	116,298.683	
	Std. Deviation	341.02593	
	Minimum	100.00	
	Maximum	1491.00	
	Range	1391.00	
	Interquartile Range	426.50	
	Skewness	1.341	.536
	Kurtosis	2.681	1.038
VA.2	Mean	19.7222	3.71519
	95% Confidence Interval for Mean	Lower Bound	11.8839
		Upper Bound	27.5606
	5% Trimmed Mean	18.0247	
	Median	17.5000	
	Variance	248.448	
	Std. Deviation	15.76222	
	Minimum	.00	
	Maximum	70.00	
	Range	70.00	
	Interquartile Range	12.50	
	Skewness	2.085	.536
	Kurtosis	5.616	1.038
VA.3	Mean	40.8333	5.33594
	95% Confidence Interval for Mean	Lower Bound	29.5755
		Upper Bound	52.0912
	5% Trimmed Mean	40.0926	
	Median	40.0000	
	Variance	512.500	
	Std. Deviation	22.63846	
	Minimum	5.00	
	Maximum	90.00	
	Range	85.00	
	Interquartile Range	32.50	
	Skewness	.378	.536
	Kurtosis	−0.028	1.038
VA.4	Mean	43.6111	5.18932
	95% Confidence Interval for Mean	Lower Bound	32.6626
		Upper Bound	54.5596
	5% Trimmed Mean	43.1790	
	Median	40.0000	
	Variance	484.722	
	Std. Deviation	22.01641	
	Minimum	5.00	
	Maximum	90.00	
	Range	85.00	
	Interquartile Range	25.00	
	Skewness	.698	.536
	Kurtosis	.169	1.038
VA.5	Mean	15.5556	1.93391
	95% Confidence Interval for Mean	Lower Bound	11.4754
		Upper Bound	19.6358
	5% Trimmed Mean	15.3395	
	Median	20.0000	
	Variance	67.320	
	Std. Deviation	8.20489	
	Minimum	5.00	
	Maximum	30.00	
	Range	25.00	
	Interquartile Range	11.25	
	Skewness	.160	.536
	Kurtosis	−0.956	1.038
VA.6	Mean	18.8889	3.09320
	95% Confidence Interval for Mean	Lower Bound	12.3628
		Upper Bound	25.4150
	5% Trimmed Mean	18.4877	
	Median	12.5000	
	Variance	172.222	
	Std. Deviation	13.12335	
	Minimum	5.00	
	Maximum	40.00	
	Range	35.00	
	Interquartile Range	25.00	
	Skewness	.316	.536
	Kurtosis	−1.634	1.038
VA.7	Mean	15.5556	3.25619
	95% Confidence Interval for Mean	Lower Bound	8.6856
		Upper Bound	22.4255
	5% Trimmed Mean	14.7840	
	Median	10.0000	
	Variance	190.850	
	Std. Deviation	13.81484	
	Minimum	5.00	
	Maximum	40.00	
	Range	35.00	
	Interquartile Range	25.00	
	Skewness	1.000	.536
	Kurtosis	−0.709	1.038
VA.8	Mean	13.0556	3.57320
	95% Confidence Interval for Mean	Lower Bound	5.5168
		Upper Bound	20.5944
	5% Trimmed Mean	11.1728	
	Median	7.5000	
	Variance	229.820	
	Std. Deviation	15.15982	
	Minimum	.00	
	Maximum	60.00	
	Range	60.00	
	Interquartile Range	10.00	
	Skewness	2.086	.536
	Kurtosis	4.627	1.038
VA.9	Mean	6.9444	1.15321
	95% Confidence Interval for Mean	Lower Bound	4.5114
		Upper Bound	9.3775
	5% Trimmed Mean	6.6049	
	Median	5.0000	
	Variance	23.938	
	Std. Deviation	4.89264	
	Minimum	.00	
	Maximum	20.00	
	Range	20.00	
	Interquartile Range	5.00	
	Skewness	.773	.536
	Kurtosis	1.762	1.038
VA.10	Mean	5.0000	.90388
	95% Confidence Interval for Mean	Lower Bound	3.0930
		Upper Bound	6.9070
	5% Trimmed Mean	5.0000	
	Median	5.0000	
	Variance	14.706	
	Std. Deviation	3.83482	
	Minimum	.00	
	Maximum	10.00	
	Range	10.00	
	Interquartile Range	10.00	
	Skewness	.000	.536
	Kurtosis	−1.190	1.038

## 2 Experimental design, materials, and methods

In summary, our ports data includes 18 different places. These ports are appearing to be consistently important places for ocean shipping. Others appear in the data in different benchmark years, which indicates real changes in use and was similar with the concept of the study by [4], but in this data has also distinct recording practices at different times and between the sources. Fig. 2 shows the aggregate distribution of the number of appearances of each variables for all ports.

Appendix A. Supplementary data

Supplementary data to this article can be found online at https://data.mendeley.com/datasets/jxj6dt54w6/1

## Declaration of Competing Interest

The authors declare that they have no known competing financial interests or personal relation-ships that could have appeared to influence the work reported in this paper.

## Appendix Supplementary materials

Image, application 1

Image, application 2
